# Cognitive Impairment in Parkinson's Disease: The Dual Syndrome Hypothesis

**DOI:** 10.1159/000341998

**Published:** 2012-10-03

**Authors:** Angie A. Kehagia, Roger A. Barker, Trevor W. Robbins

**Affiliations:** ^a^School of Psychology, University of St. Andrews, St. Andrews, UK; ^b^Cambridge Centre for Brain Repair, Department of Clinical Neurosciences, University of Cambridge, Cambridge, UK; ^c^Behavioural and Clinical Neuroscience Institute, University of Cambridge, Cambridge, UK; ^d^Department of Experimental Psychology, University of Cambridge, Cambridge, UK

**Keywords:** Acetylcholine, Catechol-o-methyl-transferase, Dementia, Dopamine, Dual syndrome hypothesis, Executive function, Fronto-striatal dysfunction, Noradrenaline, Overdose, Parkinson's disease

## Abstract

Research into the heterogeneous nature of cognitive impairment documented in patients with Parkinson's disease (PD) has focused on disentangling deficits that vary between individuals, evolve and respond differentially to pharmacological treatments, and relate differentially to PD dementia (PDD). We summarise studies conducted in our laboratory over the last 2 decades, outlining the incremental development of our hypotheses, the starting point for which is our early work on executive deficits mirroring fronto-striatal dysfunction. We present subsequent findings linking these deficits to a model of dopaminergic function that conforms to an inverted curvilinear function. We review studies that investigated the range of dopamine-independent attentional and visuospatial memory deficits seen in PD, demonstrating that abnormalities in these domains more accurately predict PDD. We conclude with an exposition of the dual syndrome hypothesis, which distinguishes between dopaminergically mediated fronto-striatal executive impairments and a dementia syndrome with distinctive prodromal visuospatial deficits in which cholinergic treatments offer some clinical benefits.

## Introduction

Parsing the heterogeneous nature of cognitive impairment in Parkinson's disease (PD) into ontologically meaningful deficits entails establishing clear cognitive, anatomical and neurochemical definitions for these deficits as independent and/or hierarchically related entities. In essence, our task has been that of interpreting neuropsychological variation between and within individual patients and groups, at a single or multiple time points, employing pharmacological manipulations, neuroimaging and analyses of genotypic variation to delineate the nature of each of these deficits.

**Fig. 1:**
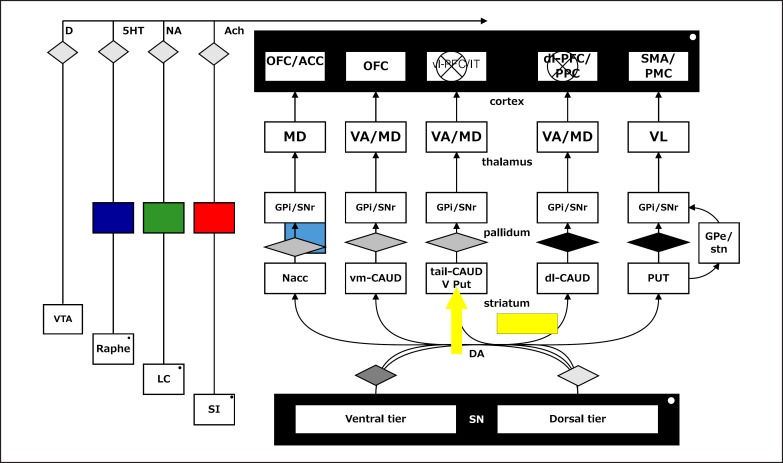
Schematic diagram of the cortico-striatal circuitry and possible causes of cognitive deficits in PD (superimposed blocks or arrows). Note possible effects on mesocortical dopamine (from the ventral tegmental area); the serotoninergic (5HT) raphé nuclei; the noradrenergic (NA) projection to the cortex from the locus coeruleus (LC) and the cholinergic input from the basal forebrain or substantia innominata (SI). Lewy bodies or associated neurodegeneration could impair cortical functioning. Following either depletion (block) or overactive (arrow) dopamine (D), systems could modulate cognitive function. ACh = Acetylcholine; OFC/ACC = orbitofrontal cortex/anterior cingulate; vl-PFC = ventrolateral PFC; PPC = posterior parietal cortex; dlPFC = dorsolateral PFC; SMA/PMC = supplementary motor area/premotor cortex; MD = mediodorsal; VA = ventral-anterior; VL = ventrolateral; GPi = globus pallidus, internal segment; SNr = substantia nigra, pars reticulata; GPe = globus pallidus, external segment; stn = subthalamic nucleus; Nacc = nucleus accumbens; vm-CAUD = ventromedial caudate nucleus; tail-CAUD = tail of the caudate; V Put = ventral putamen; dl-CAUD = dorsolateral caudate; PUT = putamen; SN = substantia nigra.

The diversity of progressive neurodegeneration in PD is secondary to the intracellular fibrillisation of α-synuclein, which is its major pathological hallmark, and additional neuropathological features may include vascular disease, Lewy bodies, neurofibrillary tangles and amyloid plaques more commonly seen in dementia. This impacts on the major neurotransmitter systems at uneven rates and asymmetrically between individuals, rendering analyses of cognitive impairment patterns particularly challenging: early nigrostriatal degeneration causes progressive loss of dopamine neurotransmission in a dorsal to ventral gradient within the basal ganglia [1], unevenly affecting different fronto-striatal ‘loops’ [2] at different stages of the disease in all patients. Parallel deficits in the mesocorticolimbic dopamine systems originating in the midbrain also develop. Beyond causing pronounced dopaminergic disturbance, PD also detrimentally affects the noradrenergic, serotoninergic and cholinergic systems, by causing degeneration respectively of the locus coeruleus [3,4,5], dorsal raphé nuclei [6,7] and cholinergic brainstem nuclei, particularly the basal nucleus of Meynert [8] (fig. 1).

The evolution and heterogeneity of cognitive impairment in PD mirrors the complexity of the disease process, in terms of the variable as well as the interacting compromise of the catecholaminergic and cholinergic systems. Mild cognitive impairment (MCI) in the form of executive and working memory deficits similar to those seen in frontal lobe-damaged patients is present in up to 50% of cases; however, PD dementia (PDD), a distinctive pattern of rapid cognitive decline leading to aphasia, apraxia and agnosia (thus resembling deficits seen in patients with temporal lobe damage and cortical dementia), is only seen in perhaps as many as 10% of cases [9]. Indeed, PDD represents a superordinate aspect of clinical heterogeneity due to its detrimental implications for patient mortality and quality of life [10]. In this review, we chart our progress over the last 2 decades using a quasi-chronological approach culminating in the dual syndrome hypothesis, which captures the evolution in our understanding of cognitive dysfunction in PD.

## Dopaminergic Amelioration of Executive Deficits Mirrors Fronto-Striatal Function

**Fig. 2:**
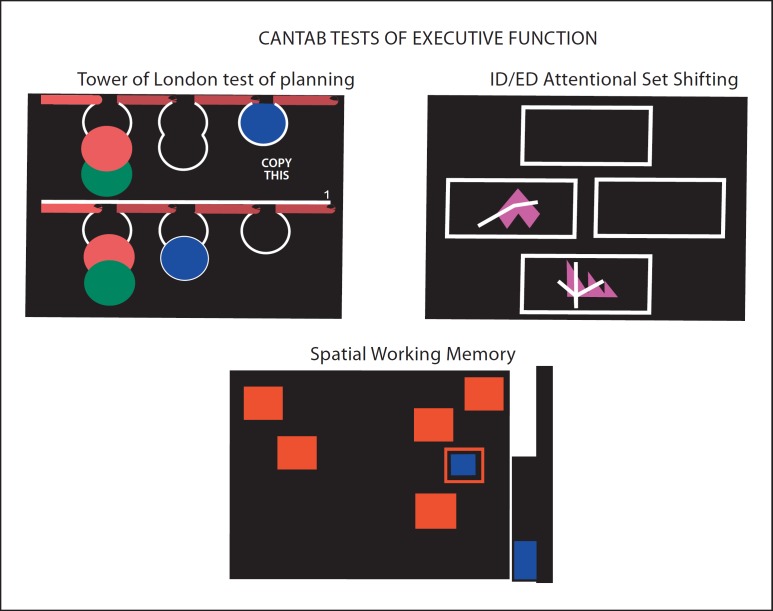
Screen shots of three of the CANTAB tests of fronto-executive function; the ‘SoC’ version of the ToL test of planning; the intra-dimensional (ID)/ED set shifting task and the self-ordered spatial working memory task – all configured to work via touch-sensitive screen methodology. These tasks were used in a study by Owen et al. [21] to define fronto-striatal cognitive deficits in PD. Reproduced with permission from Cambridge Cognition.

Early research into the cognitive impairment patterns caused by PD revealed deficits across the cognition spectrum, in terms of visuospatial memory and learning [11,12,13,14] and, prototypically, executive function including planning, problem solving and attentional shifting, on tests such as the Wisconsin Card Sorting Test and Tower of London (ToL) [15,16,17,18,19,20]. The PD dysexecutive syndrome exhibits similarities to the effects of frontal lobe damage, and its conceptualisation as a fronto-striatal deficit is well exemplified by our early studies [21,22], comparing the performance of unmedicated and medicated PD patients at different disease stages on a test battery previously shown to be sensitive to frontal lobe lesions [23]. The tests included the Stockings of Cambridge (SoC) test of planning (derived from the ToL test), intra-/extra-dimensional (ED) set shifting, which is a test of shifting aptitude between stimulus exemplars and higher-order perceptual dimensions, and the self-ordered Spatial Working Memory task of the Cambridge Neuropsychological Test Automated Battery (CANTAB; fig. 2) [24]. These studies revealed planning and spatial working memory deficits as a function of disease severity in the PD groups, which paralleled those seen in patients with frontal lobe excisions.

**Fig. 3:**
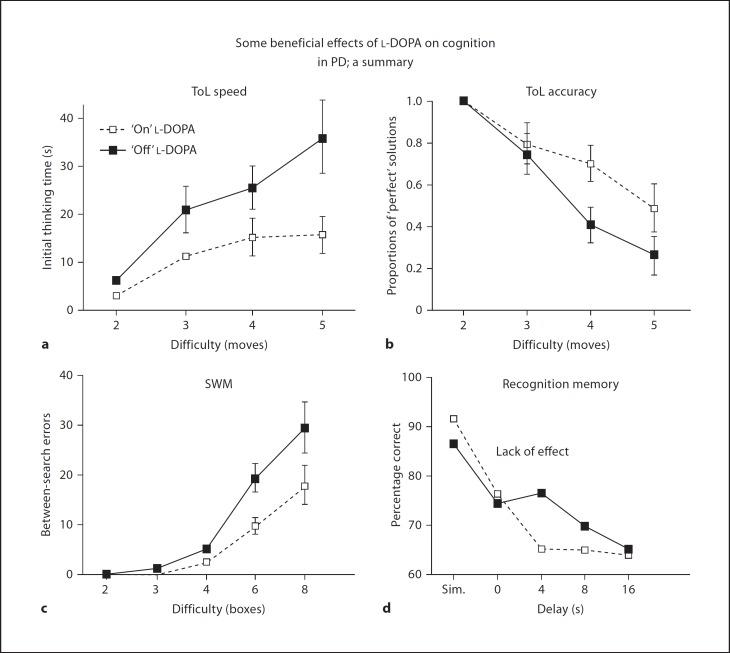
Effects of l-DOPA medication withdrawal in PD. Significant impairments are observed in ToL performance, as measured by latency (**a**) and accuracy (**b**; see also fig. 2) and self-ordered spatial working memory (SWM) performance (**c**; see also fig. 2), but not in visual recognition memory (**d**), which is impaired relative to age and IQ-matched controls. Sim. = Simultaneous matching. Reproduced from Lange et al. [25], with permission from the publishers.

Critically, we [22,25] demonstrated specific beneficial effects of dopaminergic medication on planning and spatial working memory, but not visual recognition memory (fig. 3), visuospatial paired associates learning (PAL; a delayed cued recall test) or ED set shifting, more clearly defining earlier findings of general cognitive amelioration as a result of dopaminergic pharmacotherapy [26,27] as specific improvements in executive function mirroring fronto-striatal enhancement. These findings heralded a range of studies employing short-term withdrawal from levodopa (l-DOPA), the dopamine precursor included in most therapeutic regimes. These studies extended demonstrations of dopaminergic enhancement to other tasks sensitive to fronto-striatal dysfunction, including task switching in the face of stimulus interference [28,29] and verbal working memory at the manipulation rather than the retrieval phase of the task [30]. On this verbal working memory paradigm, we found that impaired but not cognitively intact PD patients demonstrated specific reductions in dorsal fronto-striatal blood oxygenation level-dependent signal change using functional magnetic resonance imaging (fMRI) [31], further emphasizing the case that these deficits have a locus within a dopaminergic fronto-striatal network.

## Dopaminergic ‘Overdosing’ Leads to Impulsivity

**Fig. 4:**
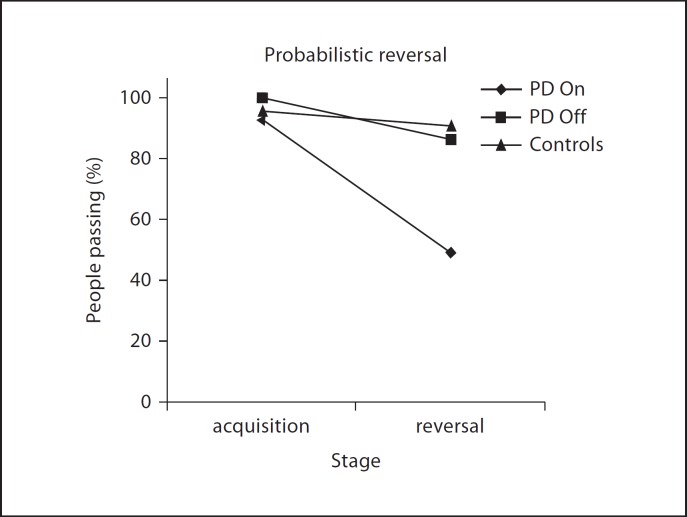
Detrimental effects on probabilistic reversal learning following l-DOPA medication in PD, according to the number of patients successfully passing a learning criterion. Note the absence of drug effects in the learning stage. Reproduced from Swainson et al. [33], with permission from the publishers.

**Fig. 5:**
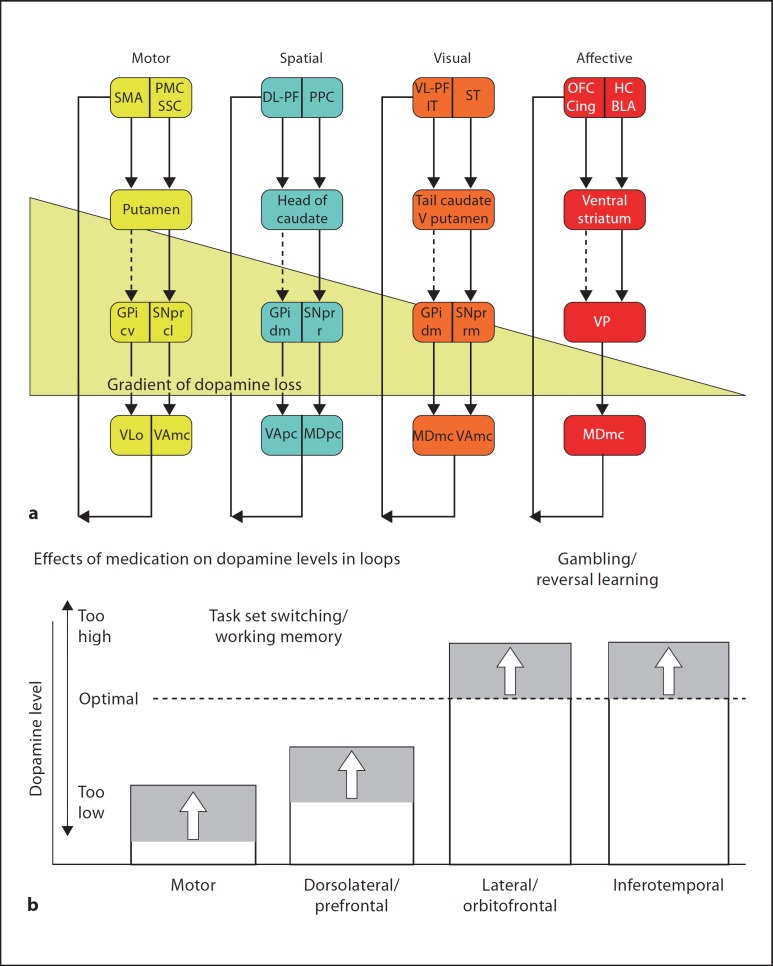
**a** Schematic depiction of four of the ‘fronto-striatal loops’, ranging from a ‘motor’ loop involving the putamen and supplementary motor area through to an ‘affective’ loop comprising the nucleus accumbens and certain areas of the orbitofrontal cortex, with two prominent ‘cognitive’ loops in between (reproduced from Lawrence et al. [36]). Also shown is the hypothesised gradient of dopamine depletion for PD which is greatest in the dorsal striatum and weakest in the ventral striatum. SMA = Supplementary motor area; SSC = somatosensory cortex; PMC = premotor cortex; PPC = posterior parietal cortex; DL-PF = dorsolateral prefrontal cortex; VL- PF = ventrolateral prefrontal cortex; ST = superior temporal cortex; OFC = orbitofrontal cortex; Cing = cingulate cortex; HC = hippocampus; BLA = basolateral amygdala; V putamen = ventral putamen; GPi = globus pallidus, internal segment; VP = ventral pallidum; SNpr = substantia nigra pars reticulata; cv = caudoventral; cl = caudolateral; dm = dorsomedial; r = rostral; rm = rostromedial; VLo = ventrolateral thalamus; VA = ventral anterior thalamus; MD = mediodorsal thalamus; mc = magnocellular; pc = parvocellular. **b** Schematic rendering of typical forms of behaviour associated with each loop after withdrawal of dopaminergic medication. Note on the extreme left, tasks such as task set switching and spatial working memory are mildly improved by dopamine medication. By contrast, on gambling and reversal learning, tasks associated with ventral frontal mediation, l-DOPA withdrawal significantly impairs performance. Reproduced with permission from Swainson et al. [33] and the publishers.

Withdrawal studies were instrumental in revealing the role of dopamine neurotransmission in the fronto-striatal circuitry in forms of cognitive impairment in PD by demonstrating not only benefits, but also pernicious effects, of dopaminergic medication. Such effects were reported initially by Gotham et al. [32] in terms of deterioration in conditional learning, alongside improvements in alternating fluency. In one of the earliest findings in support of a putative dopaminergic ‘overdosing’ theory, Swainson et al. [33] found that medicated PD patients showed a non-perseverative reversal deficit during probabilistic reversal learning similar to that seen in a group of frontal and temporal lobe lesion patients. In contrast, the l-DOPA withdrawn PD group surprisingly revealed unimpaired performance in the form of intact reversal learning (fig. 4). In a replication of this result, Cools et al. [28] demonstrated reversal deficits alongside improvements in task switching in patients tested on their normal dopaminergic regime, and the opposite pattern following withdrawal. These findings were extended to the Cambridge Gambling Task, which is sensitive to orbitofrontal damage and where l-DOPA led to increased impulsive, delay-averse behaviour in PD patients which was absent when the same patients were withdrawn from medication [29]. Both probabilistic reversal learning and the Cambridge Gambling Task rely on ventral fronto-striatal circuitry: the former is associated with blood oxygenation level-dependent signal change in the ventral striatum and inferior frontal cortex [34], and the latter with increases in regional cerebral blood flow in the orbitofrontal cortex [35]. Thus, considering the uneven gradient of dopamine loss across the corticostriatal ‘loops’ in early PD, we hypothesised that dopaminergic medication restores neurotransmission in severely affected dorsal circuits, implicated in executive deficits of planning and working memory, but putatively ‘overdoses’ relatively less affected ventral circuits implicated in reward processing and learning [28,33,36] (fig. 5). Beyond striatal effects, the dopamine overdose hypothesis may also account for diminution in reward sensitivity sometimes seen in medicated PD patients by additionally implicating the mesolimbic dopamine pathway, from which the ventral striatum and amygdala receive midbrain dopamine projections. Putatively, dopaminergic medication may cause an overall increase in tonic dopamine that obscures phasic dopamine dips evoked by error-correcting feedback [37]. An fMRI study of probabilistic reversal learning in PD patients tested ‘on’ and ‘off’ l-DOPA implicated the nucleus accumbens, but not the dorsal striatum or prefrontal cortex (PFC), as the site of this medication effect, providing support for this hypothesis [38]. A similar mechanism of action involving the ventral striatum may account for the emergence of impulse control deficits and compulsive gambling associated with dopamine agonist treatment [39], in particular pramipexole [40].

## Further Complications: Prefrontal Dopamine and Cognitive Deficits in PD

Given the tripartite division of the main midbrain dopamine pathways into the nigrostriatal, mesolimbic and mesocortical systems, it would appear reasonable to assume that the mesocortical projection is additionally implicated in aspects of cognitive impairment in PD, especially in fronto-striatal deficits of working memory and planning, consistent with preclinical evidence on the role of dopamine in normal cognitive function [41]. For instance, improvements in spatial working memory and planning conferred by l-DOPA are associated with *decreased* regional cerebral blood flow in the right dorsolateral PFC [42], suggesting that l-DOPA may enhance PFC *efficiency* in PD. However, the locus of this enhancement could well be in the caudate nucleus rather than the PFC itself, with a consequent improvement of functioning arising due to dopaminergic enhancement within the fronto-striatal ‘loops’ linking the cortex and striatum, rather than any isolated cortical or subcortical site.

Moreover, instead of an obvious loss of cortical dopamine, ^18^F-fluorodopa positron emission tomography (PET) studies have shown what appears to be a compensatory up-regulation in prefrontal cortical dopamine metabolism in early (unilateral, Hoehn and Yahr stage I) compared to later (bilateral) stage PD [43,44], possibly reflecting the reciprocal relationship it often bears to the activity of subcortical (mainly striatal) dopamine systems [45,46]. This up-regulation clearly complicates analyses of the role of PFC dopamine in parkinsonian cognition and could conceivably even mask impairments stemming from striatal dopamine loss. Presumably, however, a reduction in PFC dopamine levels eventually contributes to the emergent cognitive deficit pattern as the disease progresses from unilateral to bilateral and more severe impairment.

These hypotheses are difficult to test using conventional methods, but an opportunity to do so arose from the discovery of the catechol-o-methyl-transferase (COMT) polymorphism, for which a single methionine (Met) to valine (Val) substitution at residue 158 confers up to fourfold increase in enzymatic efficiency as a function of the number of Val alleles carried. In healthy individuals, Val homozygosity compared with heterozygosity confers greater COMT efficiency, or more effective catabolic methylation of dopamine, thereby reducing PFC dopamine levels. By contrast, Met homozygocity renders COMT *less* efficient, leading to increased PFC dopamine levels (and putatively, enhanced PFC activity). Although dopamine transporters play a predominant role in the regulation of striatal dopamine, their relative absence in the PFC renders COMT activity in this region the major means of regulating dopamine neurotransmission. A much simplified summary of the considerable literature stimulated by the findings of Weinberger and colleagues [47,48] in healthy individuals and patients with schizophrenia holds that, in general, COMT polymorphism confers superior performance on tasks sensitive to prefrontal function, such as working memory, as a function of the number of Met alleles carried. Met homozygotes outperform Val homozygotes [47,48] in a pattern that conforms to an inverted U-shaped function, analogous to the Yerkes-Dodson principle [49]. These findings bear obvious implications concerning those aspects of parkinsonian cognition mirroring prefrontal dopamine neurotransmission and raised the possibility of adopting a neurogenetic approach to further investigating these deficits.

In collaboration with Weinberger's group, and in the context of the Cambridgeshire Parkinson's Incidence from GP to Neurologist (CamPaIGN) cohort study which we elaborate on later, 288 PD patients stratified by COMT polymorphism were assessed, focusing in particular on the CANTAB SoC test [50]. Surprisingly, Met homozygosity was associated with impaired planning accuracy in PD, an effect especially magnified in male patients and those on dopaminergic medication. A subsequent fMRI study confirmed that Met-homozygote patients showed hypoactivation in regions underpinning planning performance that included the dorsolateral PFC, frontopolar and parietal cortex [51].

**Fig. 6:**
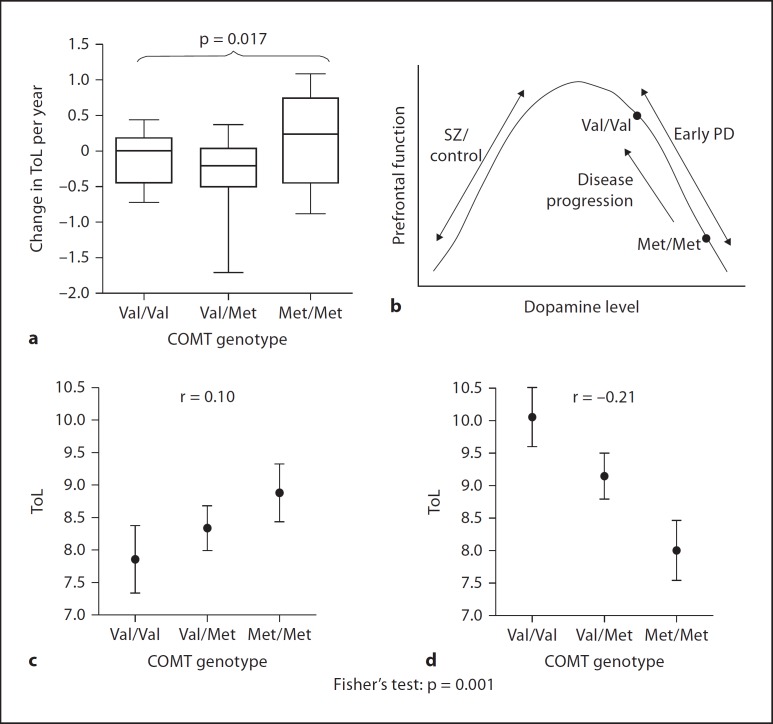
**a** Longitudinal analysis: 5-year follow-up of patients with PD (n = 70) on performance on the ToL test of planning as a function of COMT genotype. **b** Hypothesised inverted U-shaped curve explaining data in terms of the Yerkes-Dodson curve. The performance of patients with PD is contrasted with that of patients with schizophrenia (SZ). Cross-sectional analysis of ToL planning performance in patients relatively early (<1.6 years; **d**) and later (≥1.6 years; **c**) in the course of PD (n = 425), stratified by COMT genotype. Reproduced from Williams-Gray et al. [52], with permission from the publishers.

These findings were expanded upon in a separate cohort study of 425 patients [52] which confirmed the original finding of SoC impairment in those patients with more Met alleles relatively early in the course of the disease (<1.6 years), compared with those later in the course, where the relationship was actually reversed (fig. 6c). These findings are readily interpretable in terms of a dopaminergic optimum titrated according to the hypothetical inverse U-shaped function which is exceeded in early PD: Met homozygosity may actually have detrimental effects on SoC planning at a time when PFC dopamine is up-regulated, placing Val homozygotes, unusually, at an advantage. If valid, this hypothesis predicts improvements for Met homozygote patients as their disease progresses and the initially up-regulated PFC dopaminergic system eventually becomes compromised. Indeed, at the 5-year follow-up of 70 of these PD patients, the initially disadvantaged Met homozygotes actually exhibited differential *improvement* in planning performance over this time span compared with Val homozygotes (in whom no change was documented; fig. 6a) [52]. This observation implies that their initial deficit may well have been caused in part by excess PFC dopamine which was ameliorated as the disease progressed, possibly as a function of progressive loss in mesocortical dopamine.

Collectively, this series of studies represents a most unusual case of symptom *reversal* with increasing disease progression, and, crucially, has offered perhaps a unique form of endorsement of the inverted-U hypothesis (fig. 6b). This clearly supersedes earlier simplistic hypotheses that dopamine loss in PD is responsible for producing the cognitive deficits associated with the disease, and that dopaminergic medication may restore many aspects of cognitive functioning. Not only may ‘overdosing’ of the mesolimbic circuitry occur, as proposed originally by Swainson et al. [33] and Cools et al. [28], but a relative excess of PFC dopamine may additionally contribute to aspects of cognitive impairment in PD.

Overall, these findings have highlighted new facets of the PD dysexecutive syndrome and accommodate the variability in its manifestation as one which mirrors fronto-striatal modulation according to a Yerkes-Dodson-like curvilinear function, subject not only to disease stage and dopaminergic medication, but also genetically determined properties of dopamine neurotransmission.

## Beyond Dopamine: Set Shifting and Memory Deficits in PD

Over the course of this research program focusing on the dopamine-dependent aspects of cognitive impairment in PD utilizing dopaminergic withdrawal, comparisons between medicated and pharmacologically naïve patients, as well as neuroimaging and genetics, we have alluded to two emergent sets of findings which point to orthogonal patterns of impairment in PD reflecting different pharmacological and neural substrates.

The first of these concerns ED set shifting, where patients are required to shift attention not between stimuli, a capacity which is known to be sensitive to striatal dopamine, but between higher-order perceptual dimensions or modalities (e.g. from lines to shapes). PD patients exhibit reliable deficits in ED shifting [53], but dopaminergic withdrawal has no effect on this aspect of higher-order attentional control [28,30,54,55,56], or on task switching between categorical stimulus judgments [57]. These results are in line with animal work showing that striatal dopamine depletion in the marmoset produces deficits in reversal learning but not ED shifting (at least for the initial shift) [58]. Further evidence from studies employing noradrenergic de-afferentation in the rat [59,60] implicates noradrenaline in the ED shifting component of the parkinsonian executive deficit [for review, see ref. [61]] which is consistent with the early and profound degeneration of the locus coeruleus in PD [3]. Indeed, improvements in global cognition and wakefulness have been shown in an 8-week, placebo-controlled study of the noradrenaline re-uptake inhibitor atomoxetine in PD patients [62], while a smaller uncontrolled trial, using however flexible dosing of atomoxetine, demonstrated specific improvements in executive function and attention in PD [63]. Investigation of this noradrenergic hypothesis of higher-order cognition in PD is also currently underway in our laboratory.

In addition to executive function, visual memory is often [11,12,13,55] but not always impaired in PD [64]. For example, early work by Sahakian et al. [11] highlighted visual pattern and spatial recognition memory deficits in a severely affected, dopaminergically medicated patient group but not in more mildly affected unmedicated patients, while both groups were impaired on a task of visuospatial PAL. The latter deficits in PAL are significant in that this particular task has been found to predict a diagnosis of Alzheimer's disease in a group of patients with questionable dementia (MCI) recruited on the basis of subjective memory complaints in a memory clinic [65]. In two follow-up studies, we addressed the role of dopaminergic medication in recognition memory. Employing l-DOPA withdrawal within subjects, Lange et al. [25] demonstrated that withdrawal caused a deterioration in spatial working memory and ToL performance, but had no effect on tests of visual pattern (fig. 3) and spatial recognition memory, visuospatial associative learning as well as simultaneous and visual delayed-matching-to-sample tasks. Consistent with this and using the same tasks, Owen et al. [55] found no differences as a function of medication status between medication-naïve and mildly medicated patients, reporting deficits only in the more severely affected medicated group. These aspects of the PD mnemonic deficit stand in contrast to dopamine-dependent working memory, which relies on online, self-ordered manipulation of stimulus representations. Instead, these impairment patterns quite possibly reflect cholinergic deficits in the frontal and temporal cortex, in agreement with known pathology in the basal forebrain cholinergic nuclei and ascending pathways to the frontal and temporal cortices present even at early disease stages [66]. This interpretation gains support from observations of memory deficits, particularly at short retention intervals, indicating deficits during registration, following long-term administration of anti-cholinergic medication in PD [67], as well as detrimental effects on memory caused by the muscarinic antagonist hyoscine (scopolamine) in a group of PD patients, but not in a matched control group [68]. Treatment with another muscarinic antagonist, trihexiphenidyl, has also been associated with PD impairments on a visual associative learning task of transfer generalisation [69].

## Contributions from Longitudinal Cohort Studies to Understanding Clinical Heterogeneity

Most studies investigating the nature of cognitive dysfunction in PD employ prevalent cross-sectional cohorts of patients, usually screened for the presence of dementia using the Mini-Mental State Examination (MMSE) [70]. Such studies are thus inherently biased in terms of patient selection, which limits their potential to investigate more severe cognitive impairment in the form of dementia.

Large cohort studies have been instrumental in efforts to systematically address this aspect of cognitive heterogeneity. Using a community-based epidemiological approach in a population of approximately 700,000 over a 25-month incidence period, the CamPaIGN study was the first to assess the incidence of PD and parkinsonism, and the extent and natural history of cognitive deficits in the ensuing patient cohort [50]. We identified five subgroups of patients at disease presentation, who were impaired on (i) ToL, indicating fronto-striatal deficits, (ii) visual pattern recognition memory, indicating temporal lobe dysfunction, (iii) both ToL and pattern recognition memory, as well as (iv) a cognitively preserved subgroup and (v) a group of patients who demonstrated marked cognitive impairment and had an MMSE score <24, indicating dementia.

**Fig. 7:**
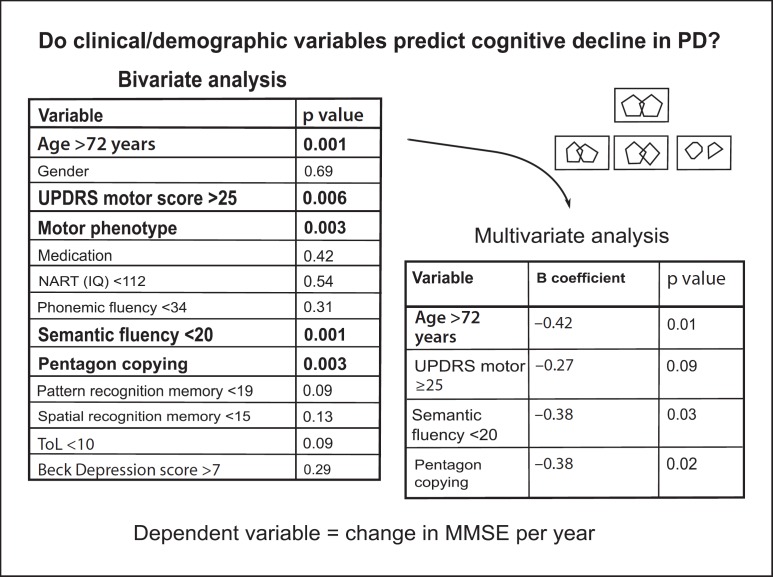
Predictive power of clinico-pathological variables for dementia in PD; pentagon copying and semantic verbal fluency from the MMSE were the best predictors. Reproduced from Williams-Gray et al. [52], with permission from the publishers. UPDRS = Unified Parkinson's Disease Rating Scale; NART = National Adult Reading Test.

In further studies on this patient cohort, we employed a data-driven approach using cluster analysis to separate patients into groups characterised by internal cohesion and external isolation [71], as well as longitudinal assessment [9] to assess the evolution of cognitive impairment. Both approaches identified a cluster of patients with mild cognitive deficits and rapid disease progression, and another cluster of cognitively impaired patients with a non-tremor dominant, akinetic motor phenotype at presentation with dopamine-unresponsive gait disturbance, potentially indicating the impact of cholinergic deficits due to degeneration of the pedunculopontine nucleus [72]. The CamPaIGN study produced a striking finding of significant clinical value given its prognostic implications: it identified a patient phenotype indicative of early transition to PDD, which was older (>72 years) at presentation and performed poorly on two simple bedside tests of semantic fluency and copying overlapping pentagons from the MMSE [9] (fig. 7).

Within the framework of the CamPaIGN study, and in parallel to our neurogenetic investigations into COMT polymorphism, we addressed genetic susceptibility factors associated with cortical Lewy body pathology that characterises PDD, namely α-synuclein and tau. Using this candidate gene approach, Goris et al. [73] found a robust association between the MAPT (microtubule-associated protein tau) gene and the rate of dementia in the incident cohort at the 3.5-year follow-up. In the CamPaIGN cohort at the 5-year follow-up and an additional cross-sectional prevalent cohort, Williams-Gray et al. [52] replicated the predictive utility of neuropsychological assessment of pentagon copying and semantic fluency, reflecting temporal lobe function, but not phonemic fluency, sensitive to frontal lobe damage, in dementia. By contrast, some of the ‘classic’ deficits within the fronto-striatal cluster, for example, on SoC (i.e. ToL) planning, were not predictive of progression to dementia, suggesting at least two independent aspects of the cognitive deficit syndrome. We also showed that the H1/H1 MAPT haplotype, but not COMT, independently predicted dementia onset.

## The Dual Syndrome Hypothesis

Our approach to understanding the heterogeneity of cognitive impairment in PD has employed a range of techniques in a large number of studies spanning 2 decades. We have integrated these diverse findings along with clinicopathological and diagnostic criteria in a comprehensive review [74], which permits a broad dichotomy to be drawn between hypothetically independent but partially overlapping syndromes of MCI and dementia in PD.

**Fig. 8:**
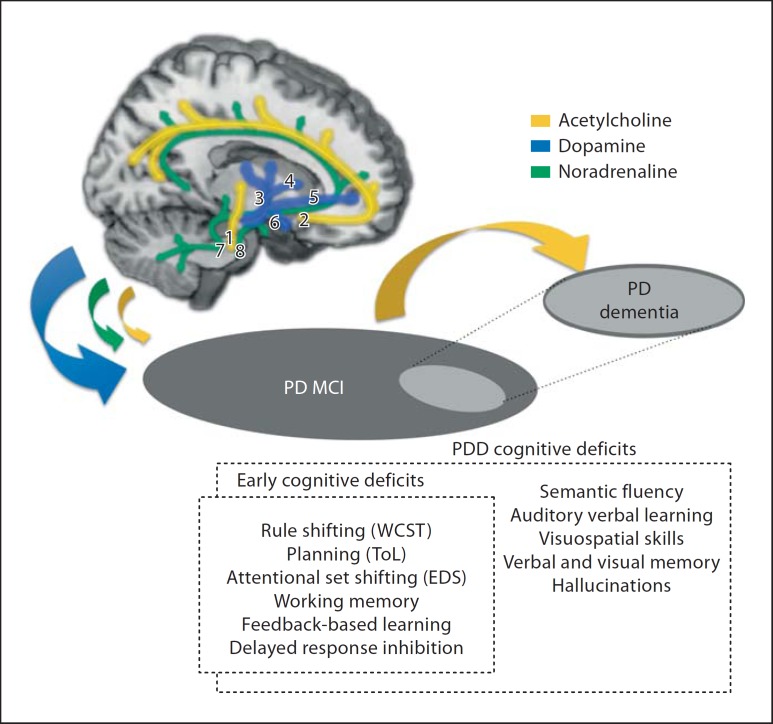
Dual syndrome hypothesis (Kehagia et al. [61,74]). The dopamine-sensitive fronto-striatal profile of deficit in PD (MCI) is contrasted with that seen in PD dementia, which may respond to a greater degree to cholinergic therapy. Pathways on the brain cut-out are those compromised by the disease and likely implicated in cognitive impairment. Cholinergic pathways: (1) from the pedunculopontine nucleus to the thalamus, (2) from the basal nucleus of Meynert to neocortex; dopaminergic pathways: (3) nigrostriatal: from the substantia nigra (pars compacta) to the striatum, (4) mesolimbic: from the ventral tegmental area to the nucleus accumbens, (5) mesocortical: from the ventral tegmental area to the frontal cortex, (6) tuberoinfundibular: from the hypothalamus to the pituitary, and noradrenergic pathways: (7) from the lateral tegmental nucleus to the amygdala and hippocampus, (8) from the locus coeruleus to the hypothalamus, thalamus, amygdala, cortex and cerebellum. WCST = Wisconsin Card Sorting Test; EDS = ED shifting.

The dual syndrome hypothesis differentiates between two broad syndromes (fig. 8): (i) a profile of neuropsychological deficit in non-demented PD patients with MCI and a tremor-dominant phenotype on tests of planning, working memory and executive function reflecting fronto-striatal dysfunction, amenable to dopaminergic amelioration but susceptible to overdosing effects, and modulated by the effects of COMT polymorphism and disease severity, and (ii) an akinetic subgroup with pronounced gait disturbance demonstrating early deficits in visuospatial function and semantic fluency indicative of posterior cortical and temporal lobe dysfunction, who exhibits rapid cognitive decline to dementia and in whom cholinergic treatment may offer some clinical benefit [75].

In addition to profound executive, attentional, mnemonic and visuospatial disturbance, PDD encompasses fluctuating attention and a range of additional psychiatric symptoms, including hallucinations and depression. Its pathological characteristics, in terms of increased Lewy body load in temporal regions and the presence of plaques and vascular changes, would appear distinct from those of idiopathic PD. However, the nosology of PDD is controversial and it remains unclear whether it lies further along the disease severity continuum in relation to PD or whether it represents a distinct clinical entity (reviewed by Kehagia et al. [74]). Nonetheless, the core features of PDD are thought to reflect widespread cholinergic dysfunction: acetylcholinesterase activity as measured by PET was found to be lower in PDD compared to non-demented PD and Alzheimer's disease patients throughout the entire cortex, and in particular in the frontal, parietal and superior temporal cortex as well as the hippocampus [76]. Moreover, consistent with the detrimental effects of anti-cholinergic medication on visuospatial memory in early PD reviewed above, cholinesterase inhibitors such as rivastigmine [75] are the most efficacious in conferring moderate clinical benefits in PDD [see [74] for discussion of possible mechanisms].

Whilst we have proposed that these ‘syndromes’, dopaminergic fronto-striatal impairments in PD on the one hand and the distinctive cholinergic visuospatial, memory and psychiatric deficits of PDD on the other, may be distinguishable, we nevertheless acknowledge some degree of overlap. PD dementia cannot exist in isolation of significant disruption in dopamine neurotransmission, given that nigrostriatal degeneration underlies the diagnosis of PD in the first instance. While mesolimbic and mesocortical dopamine dysfunction also contributes in different ways to the executive impairment profile, it should be noted that the mesocortical dopamine system innervates areas of the parietal and temporal lobes also [77,78], and, as such, could conceivably contribute to posterior cortical deficits. However, against this possibility is the lack of therapeutic response to dopaminergic medications for these typical parietal and temporal lobe impairments.

Notwithstanding the extensive evidence supporting the role of dopamine in the emergence of fronto-striatal deficits, these may also be affected by cholinergic or by other non-dopamine neurotransmitter loss due to the pervasive and interacting patterns of neurodegeneration. Some degree of frontal cholinergic compromise must also contribute to executive dysfunction, and a parallel noradrenergic imbalance may contribute to a different subset of attentional impairment. Overall, we submit that the use of the terms ‘fronto-striatal’, ‘parietal’ and ‘temporal’ lobe deficits is essentially a short-hand description for deficits that may arise also from the disconnection of neural networks involving all of these structures. Nevertheless, these convergent lines of evidence represent strong grounds for the implication of different neurocognitive systems in the profile of parkinsonian cognitive impairment, with some clear therapeutic implications.

## Conclusion

In the course of deciphering cognitively and neurochemically specific entities within the broad and heterogeneous constellation of the cognitive deficits caused by PD, we have operated within and across these levels of analysis, employing neuropsychological, pharmacological, imaging, genetic and epidemiological approaches to test constantly evolving hypotheses. We have presented these, as they evolved, beginning with early conceptualisations of the fronto-striatal syndrome and leading onto dopamine-specific hypotheses pertaining to overdosing healthy circuits and the inverted curvilinear function that may characterise dopaminergic optima as a function of COMT and disease progression. Finally, the dual syndrome hypothesis encompasses the broader range of dopamine-independent, cholinergic deficits and represents a starting point for future studies, especially given the gradual emergence of genetic risk factors. For example, could visuospatial and memory deficits seen early in the course of PD represent prodromal dementia signs? Can the dual syndrome hypothesis lead to refined functional biomarkers? We are currently investigating these questions in our ongoing ICICLE (Incidence of Cognitive Impairment in Cohorts with Longitudinal Evaluation) study, which is longitudinally following two independent incident cohorts from a population denominator of 1 million. Combining anatomic-clinical, biochemical and genetic approaches with neuropsychology and neuroimaging, we seek to identify putative biomarkers and establish a panel of tests predictive of dementia that will ultimately lead to a powerful diagnostic algorithm at the service of clinicians and research into disease-modifying agents. Thus, our hypotheses aim to explain and predict in order to contribute to disease management tailored to the needs of the individual patient. It is hoped that such knowledge will lead to improved therapies and, with this, better quality of life for patients with PD.

## Disclosure Statement

T.W.R. consults for Cambridge Cognition.

## References

[1] Kish SJ, Shannak K, Hornykiewicz O (1988). Uneven pattern of dopamine loss in the striatum of patients with idiopathic Parkinson's disease. Pathophysiologic and clinical implications. N Engl J Med.

[2] Alexander GE, DeLong MR, Strick PL (1986). Parallel organization of functionally segregated circuits linking basal ganglia and cortex. Annu Rev Neurosci.

[3] Cash R, Dennis T, L'Heureux R, Raisman R, Javoy-Agid F, Scatton B (1987). Parkinson's disease and dementia: norepinephrine and dopamine in locus ceruleus. Neurology.

[4] Chan-Palay V, Asan E (1989). Alterations in catecholamine neurons of the locus coeruleus in senile dementia of the Alzheimer type and in Parkinson's disease with and without dementia and depression. J Comp Neurol.

[5] Zarow C, Lyness SA, Mortimer JA, Chui HC (2003). Neuronal loss is greater in the locus coeruleus than nucleus basalis and substantia nigra in Alzheimer and Parkinson diseases. Arch Neurol.

[6] Brooks DJ, Piccini P (2006). Imaging in Parkinson's disease: the role of monoamines in behavior. Biol Psychiatry.

[7] Scatton B, Javoy-Agid F, Rouquier L, Dubois B, Agid Y (1983). Reduction of cortical dopamine, noradrenaline, serotonin and their metabolites in Parkinson's disease. Brain Res.

[8] Jellinger KA (1991). Pathology of Parkinson's disease. Changes other than the nigrostriatal pathway. Mol Chem Neuropathol.

[9] Williams-Gray CH, Foltynie T, Brayne CE, Robbins TW, Barker RA (2007). Evolution of cognitive dysfunction in an incident Parkinson's disease cohort. Brain.

[10] Levy G, Tang MX, Louis ED, Cote LJ, Alfaro B, Mejia H (2002). The association of incident dementia with mortality in PD. Neurology.

[11] Sahakian BJ, Morris RG, Evenden JL, Heald A, Levy R, Philpot M (1988). A comparative study of visuospatial memory and learning in Alzheimer-type dementia and Parkinson's disease. Brain.

[12] Loranger AW, Goodell H, McDowell FH, Lee JE, Sweet RD (1972). Intellectual impairment in Parkinson's syndrome. Brain.

[13] Mortimer JA, Pirozzolo FJ, Hansch EC, Webster DD (1982). Relationship of motor symptoms to intellectual deficits in Parkinson disease. Neurology.

[14] Mayeux R, Stern Y, Rosen J, Leventhal J (1981). Depression, intellectual impairment, and Parkinson disease. Neurology.

[15] Taylor AE, Saint-Cyr JA, Lang AE (1986). Frontal lobe dysfunction in Parkinson's disease: the cortical focus of neostriatal outflow. Brain.

[16] Morris RG, Downes JJ, Sahakian BJ, Evenden JL, Heald A, Robbins TW (1988). Planning and spatial working memory in Parkinson's disease. J Neurol Neurosurg Psychiatry.

[17] Robbins TW, James M, Owen AM, Lange KW, Lees AJ, Leigh PN (1994). Cognitive deficits in progressive supranuclear palsy, Parkinson's disease, and multiple system atrophy in tests sensitive to frontal lobe dysfunction. J Neurol Neurosurg Psychiatry.

[18] Canavan AG, Passingham RE, Marsden CD, Quinn N, Wyke M, Polkey CE (1990). Prism adaptation and other tasks involving spatial abilities in patients with Parkinson's disease, patients with frontal lobe lesions and patients with unilateral temporal lobectomies. Neuropsychologia.

[19] Cools AR (1984). Basal ganglia and Parkinson's disease: neurobiological and pharmacological aspects in animals and man. Clin Neurol Neurosurg.

[20] Brown RG, Marsden CD (1988). An investigation of the phenomenon of ‘set’ in Parkinson's disease. Mov Disord.

[21] Owen AM, James M, Leigh PN, Summers BA, Marsden CD, Quinn NP (1992). Fronto-striatal cognitive deficits at different stages of Parkinson's disease. Brain.

[22] Owen AM, Sahakian BJ, Hodges JR, Summers BA, Polkey CE, Robbins TW (1995). Dopamine-dependent frontostriatal planning deficits in early Parkinson's disease. Neuropsychology.

[23] Owen AM, Downes JJ, Sahakian BJ, Polkey CE, Robbins TW (1990). Planning and spatial working memory following frontal lobe lesions in man. Neuropsychologia.

[24] Robbins TW, James M, Owen AM, Sahakian BJ, McInnes L, Rabbitt P (1994). Cambridge Neuropsychological Test Automated Battery (CANTAB): a factor analytic study of a large sample of normal elderly volunteers. Dementia.

[25] Lange KW, Robbins TW, Marsden CD, James M, Owen AM, Paul GM (1992). l-dopa withdrawal in Parkinson's disease selectively impairs cognitive performance in tests sensitive to frontal lobe dysfunction. Psychopharmacology (Berl).

[26] Loranger AW, Goodell H, Lee JE, McDowell F (1972). Levodopa treatment of Parkinson's syndrome. Improved intellectual functioning. Arch Gen Psychiatry.

[27] Bowen FP, Kamienny RS, Burns MM, Yahr M (1975). Parkinsonism: effects of levodopa treatment on concept formation. Neurology.

[28] Cools R, Barker RA, Sahakian BJ, Robbins TW (2001). Enhanced or impaired cognitive function in Parkinson's disease as a function of dopaminergic medication and task demands. Cereb Cortex.

[29] Cools R, Barker RA, Sahakian BJ, Robbins TW (2003). l-Dopa medication remediates cognitive inflexibility, but increases impulsivity in patients with Parkinson's disease. Neuropsychologia.

[30] Lewis SJ, Slabosz A, Robbins TW, Barker RA, Owen AM (2005). Dopaminergic basis for deficits in working memory but not attentional set-shifting in Parkinson's disease. Neuropsychologia.

[31] Lewis SJ, Dove A, Robbins TW, Barker RA, Owen AM (2003). Cognitive impairments in early Parkinson's disease are accompanied by reductions in activity in frontostriatal neural circuitry. J Neurosci.

[32] Gotham AM, Brown RG, Marsden CD (1988). ‘Frontal’ cognitive function in patients with Parkinson's disease ‘on’ and ‘off’ levodopa. Brain.

[33] Swainson R, Rogers RD, Sahakian BJ, Summers BA, Polkey CE, Robbins TW (2000). Probabilistic learning and reversal deficits in patients with Parkinson's disease or frontal or temporal lobe lesions: possible adverse effects of dopaminergic medication. Neuropsychologia.

[34] Cools R, Clark L, Owen AM, Robbins TW (2002). Defining the neural mechanisms of probabilistic reversal learning using event-related functional magnetic resonance imaging. J Neurosci.

[35] Rogers RD, Owen AM, Middleton HC, Williams EJ, Pickard JD, Sahakian BJ (1999). Choosing between small, likely rewards and large, unlikely rewards activates inferior and orbital prefrontal cortex. J Neurosci.

[36] Lawrence AD, Sahakian BJ, Robbins TW (1998). Cognitive functions and corticostriatal circuits: insights from Huntington's disease. Trends Cogn Sci.

[37] Schultz W (2002). Getting formal with dopamine and reward. Neuron.

[38] Cools R, Lewis SJ, Clark L, Barker RA, Robbins TW (2007). l-DOPA disrupts activity in the nucleus accumbens during reversal learning in Parkinson's disease. Neuropsychopharmacology.

[39] Bostwick JM, Hecksel KA, Stevens SR, Bower JH, Ahlskog JE (2009). Frequency of new-onset pathologic compulsive gambling or hypersexuality after drug treatment of idiopathic Parkinson disease. Mayo Clin Proc.

[40] van Eimeren T, Ballanger B, Pellecchia G, Miyasaki JM, Lang AE, Strafella AP (2009). Dopamine agonists diminish value sensitivity of the orbitofrontal cortex: a trigger for pathological gambling in Parkinson's disease?. Neuropsychopharmacology.

[41] Robbins TW, Arnsten AF (2009). The neuropsychopharmacology of fronto-executive function: monoaminergic modulation. Annu Rev Neurosci.

[42] Cools R, Stefanova E, Barker RA, Robbins TW, Owen AM (2002). Dopaminergic modulation of high-level cognition in Parkinson's disease: the role of the prefrontal cortex revealed by PET. Brain.

[43] Kaasinen V, Nurmi E, Bruck A, Eskola O, Bergman J, Solin O (2001). Increased frontal [(18)F]fluorodopa uptake in early Parkinson's disease: sex differences in the prefrontal cortex. Brain.

[44] Rakshi JS, Uema T, Ito K, Bailey DL, Morrish PK, Ashburner J (1999). Frontal, midbrain and striatal dopaminergic function in early and advanced Parkinson's disease. A 3D [(18)F]dopa-PET study. Brain.

[45] Pycock CJ, Kerwin RW, Carter CJ (1980). Effect of lesion of cortical dopamine terminals on subcortical dopamine receptors in rats. Nature.

[46] Roberts AC, De Salvia MA, Wilkinson LS, Collins P, Muir JL, Everitt BJ (1994). 6-Hydroxydopamine lesions of the prefrontal cortex in monkeys enhance performance on an analog of the Wisconsin Card Sort Test: possible interactions with subcortical dopamine. J Neurosci.

[47] Egan MF, Goldberg TE, Kolachana BS, Callicott JH, Mazzanti CM, Straub RE (2001). Effect of COMT Val108/158 Met genotype on frontal lobe function and risk for schizophrenia. Proc Natl Acad Sci USA.

[48] Mattay VS, Goldberg TE, Fera F, Hariri AR, Tessitore A, Egan MF (2003). Catechol O-methyltransferase val158-met genotype and individual variation in the brain response to amphetamine. Proc Natl Acad Sci USA.

[49] Yerkes RM, Dodson JD (1908). The relation of strength of stimulus to rapidity of habit-formation. J Comp Neurol Psychol.

[50] Foltynie T, Brayne CE, Robbins TW, Barker RA (2004). The cognitive ability of an incident cohort of Parkinson's patients in the UK. The CamPaIGN study. Brain.

[51] Williams-Gray CH, Hampshire A, Robbins TW, Owen AM, Barker RA (2007). Catechol O-methyltransferase Val158Met genotype influences frontoparietal activity during planning in patients with Parkinson's disease. J Neurosci.

[52] Williams-Gray CH, Evans JR, Goris A, Foltynie T, Ban M, Robbins TW (2009). The distinct cognitive syndromes of Parkinson's disease: 5 year follow-up of the CamPaIGN cohort. Brain.

[53] Downes JJ, Roberts AC, Sahakian BJ, Evenden JL, Morris RG, Robbins TW (1989). Impaired extra-dimensional shift performance in medicated and unmedicated Parkinson's disease: evidence for a specific attentional dysfunction. Neuropsychologia.

[54] Slabosz A, Lewis SJ, Smigasiewicz K, Szymura B, Barker RA, Owen AM (2006). The role of learned irrelevance in attentional set-shifting impairments in Parkinson's disease. Neuropsychology.

[55] Owen AM, Beksinska M, James M, Leigh PN, Summers BA, Marsden CD (1993). Visuospatial memory deficits at different stages of Parkinson's disease. Neuropsychologia.

[56] Owen AM, Roberts AC, Hodges JR, Summers BA, Polkey CE, Robbins TW (1993). Contrasting mechanisms of impaired attentional set-shifting in patients with frontal lobe damage or Parkinson's disease. Brain.

[57] Kehagia AA, Cools R, Barker RA, Robbins TW (2009). Switching between abstract rules reflects disease severity but not dopaminergic status in Parkinson's disease. Neuropsychologia.

[58] Collins P, Wilkinson LS, Everitt BJ, Robbins TW, Roberts AC (2000). The effect of dopamine depletion from the caudate nucleus of the common marmoset (Callithrix jacchus) on tests of prefrontal cognitive function. Behav Neurosci.

[59] Tait DS, Brown VJ, Farovik A, Theobald DE, Dalley JW, Robbins TW (2007). Lesions of the dorsal noradrenergic bundle impair attentional set-shifting in the rat. Eur J Neurosci.

[60] McGaughy J, Ross RS, Eichenbaum H (2008). Noradrenergic, but not cholinergic, deafferentation of prefrontal cortex impairs attentional set-shifting. Neuroscience.

[61] Kehagia AA, Murray GK, Robbins TW (2010). Learning and cognitive flexibility: frontostriatal function and monoaminergic modulation. Curr Opin Neurobiol.

[62] Weintraub D, Mavandadi S, Mamikonyan E, Siderowf AD, Duda JE, Hurtig HI (2010). Atomoxetine for depression and other neuropsychiatric symptoms in Parkinson disease. Neurology.

[63] Marsh L, Biglan K, Gerstenhaber M, Williams JR (2009). Atomoxetine for the treatment of executive dysfunction in Parkinson's disease: a pilot open-label study. Mov Disord.

[64] Flowers KA, Pearce I, Pearce JM (1984). Recognition memory in Parkinson's disease. J Neurol Neurosurg Psychiatry.

[65] Blackwell AD, Sahakian BJ, Vesey R, Semple JM, Robbins TW, Hodges JR (2004). Detecting dementia: novel neuropsychological markers of preclinical Alzheimer's disease. Dement Geriatr Cogn Disord.

[66] Dubois B, Ruberg M, Javoy-Agid F, Ploska A, Agid Y (1983). A subcortico-cortical cholinergic system is affected in Parkinson's disease. Brain Res.

[67] Cooper JA, Sagar HJ, Doherty SM, Jordan N, Tidswell P, Sullivan EV (1992). Different effects of dopaminergic and anticholinergic therapies on cognitive and motor function in Parkinson's disease. A follow-up study of untreated patients. Brain.

[68] Dubois B, Danze F, Pillon B, Cusimano G, Lhermitte F, Agid Y (1987). Cholinergic-dependent cognitive deficits in Parkinson's disease. Ann Neurol.

[69] Herzallah MM, Mustafa AA, Misk AJ, Al-Dweib LH, Abdelrazeq SA, Myers CE (2010). Depression impairs learning whereas anticholinergics impair transfer generalization in Parkinson patients tested on dopaminergic medications. Cogn Behav Neurol.

[70] Folstein MF, Folstein SE, McHugh PR (1975). ‘Mini Mental State’: a practical method for grading the cognitive state of patients for the clinician. J Psychiatr Res.

[71] Lewis SJ, Foltynie T, Blackwell AD, Robbins TW, Owen AM, Barker RA (2005). Heterogeneity of Parkinson's disease in the early clinical stages using a data driven approach. J Neurol Neurosurg Psychiatry.

[72] Lewis SJ, Barker RA (2009). Understanding the dopaminergic deficits in Parkinson's disease: insights into disease heterogeneity. J Clin Neurosci.

[73] Goris A, Williams-Gray CH, Clark GR, Foltynie T, Lewis SJ, Brown J (2007). Tau and alpha-synuclein in susceptibility to, and dementia in, Parkinson's disease. Ann Neurol.

[74] Kehagia AA, Barker RA, Robbins TW (2010). Neuropsychological and clinical heterogeneity of cognitive impairment and dementia in patients with Parkinson's disease. Lancet Neurol.

[75] Emre M, Aarsland D, Albanese A, Byrne EJ, Deuschl G, De Deyn PP (2004). Rivastigmine for dementia associated with Parkinson's disease. N Engl J Med.

[76] Bohnen NI, Kaufer DI, Ivanco LS, Lopresti B, Koeppe RA, Davis JG (2003). Cortical cholinergic function is more severely affected in parkinsonian dementia than in Alzheimer disease: an in vivo positron emission tomographic study. Arch Neurol.

[77] Bentivoglio M, Morelli M, Dunnett DB, Bjorklund A, Hokfelt T (2005). The organization and circuitry of mesencephalic dopamine neurons and the distribution of dopamine receptors in the brain. The Handbook of Chemical Neuroanatomy.

[78] De Keyser J, Ebinger G, Vauquelin G (1989). Evidence for a widespread dopaminergic innervation of the human cerebral neocortex. Neurosci Lett.

